# De Novo Lower Urinary Tract Symptoms in COVID-19 Patients

**DOI:** 10.7759/cureus.33947

**Published:** 2023-01-18

**Authors:** Saurabh Jain, Akanksha Kothari, Dharmendra K Pipal, Vibha Rani, Seema Yadav, Vinay Tomar, Mukteshwar Kumar, Anupam Bhargava, Amir Usmani, Amit Soni

**Affiliations:** 1 Urology, Sawai Man Singh Medical College, Jodhpur, IND; 2 Emergency Medicine and Anaesthesiology, Jaipur National University Institute for Medical Sciences and Research Centre, Jaipur, IND; 3 General, Colorectal and Minimal Access Surgery, All India Institute of Medical Sciences, Gorakhpur, Gorakhpur, IND; 4 Gynaecology and Obstetrics, All India Institute of Medical Sciences, Gorakhpur, Gorakhpur, IND; 5 Anaesthesia, Jaipur National University Institute for Medical Sciences and Research Centre, Jaipur, IND; 6 Urology, Sawai Man Singh Medical College, Jaipur, IND; 7 Urology and Renal Transplant, Dr. Ram Manohar Lohia Institute of Medical Sciences, Lucknow, IND; 8 Genito-Urinary Surgery, Sri Venkateswara Institute of Medical Sciences, Tirupati, IND; 9 Urology, Sanjay Gandhi Post Graduate Institute of Medical Sciences, Lucknow, IND; 10 Urology, Govt. Medical College, Kota, IND

**Keywords:** covid-19, luts, storage symptoms, computerised tomography (ct) score, overactive bladder (oab) score

## Abstract

Background and objective

Since early 2020, the novel coronavirus disease 2019 (COVID-19) has turned into a global healthcare concern. The usual clinical presentation of COVID-19 infection includes myalgia, headache associated with pyrexia, and sore throat. Our study aimed to assess the severity of lower urinary tract symptoms (LUTS) in COVID-19 patients and determine its correlation with the prognosis of the disease.

Methods

We conducted an observational study in the COVID-19 care unit at a tertiary care teaching center in Rajasthan on patients diagnosed as COVID-19-positive. The overactive bladder (OAB) symptom scoring system for LUTS and the CT scoring system for lung involvement in COVID-19 patients were used to evaluate the sample population.

Results

While our findings showed a non-significant association between OAB and CT score (p>0.05), correlation analysis revealed that the length of hospital stay was significantly longer and oxygen needs were significantly more frequent with severe LUTS.

Conclusions

Based on our findings, de novo LUTS, particularly storage symptoms, may be present in COVID-19-positive cases, and the severity of these symptoms may have an impact on the patient's length of stay in the hospital. Hence, doctors and other medical professionals should consider COVID-19-related bladder dysfunctions such as de novo LUTS as part of COVID-19 symptomatology.

## Introduction

The emergence of coronavirus disease 2019 (COVID-19), caused by the severe acute respiratory syndrome coronavirus (SARS-CoV-2), first notified by Chinese authorities on December 31, 2019, has presented an unprecedented challenge for healthcare systems globally. Due to its high infectivity, the disease has rapidly spread across the world in a very small period. The first case of COVID-19 was reported in the Wuhan province of China [1]. Because of the exponential global transmission of the virus, the World Health Organization (WHO) declared COVID-19 a Public Health Emergency of International Concern on 30 January 2020, and a global pandemic on March 11, 2020 [2].

Clinically, the presentation of COVID-19 infection ranges from being asymptomatic to mild to severe symptoms. Symptoms such as myalgia, fever, and headache are common in COVID-19 patients. Sore throat, diarrhea, nausea and diarrhea, loss of smell and taste, and abdominal pain are some of the other symptoms. Thus, the dominant clinical manifestation of COVID-19 is respiratory in nature, ranging from a mild flu-like illness to lethal and often fatal acute respiratory distress [3].

Along with commonly reported symptoms, the emergence of several new symptoms involving other organs has been reported. COVID-19 may manifest with classic urinary symptoms without any urosepsis, which may complicate and create a dilemma in the differential diagnosis for urologists [4]. It was observed that patients had higher urinary frequency without urinary tract infections. The increased presence of lower urinary tract symptoms (LUTS) in COVID-19-positive individuals without any indication of bacterial infection or prostatitis may be due to viral invasion [5].

Recent molecular studies have shown that the COVID-19 spike protein shows a firm binding affinity for angiotensin-converting enzyme 2 (ACE2) receptors and is crucial for the entry of SARS-CoV-2 into the human cell. Thus, it is stipulated that organs expressing ACE2 receptors, such as the kidney (proximal convoluted tubules), testis, and bladder, can be at risk of damage by COVID-19 [6].

Zou et al. demonstrated a cut-off estimation or value for the proportion of ACE2 receptor expression in different organs [7]. The presence of ACE2 receptors in the urothelial cells is up to 2.4%, which may enable the entry of the COVID-19 virus, causing viral cystitis. Additionally, the existence of viral RNA in the urine supports the correlation of COVID-19 infection with LUTS [8]. In addition to the infection of urothelial cells causing cystitis, irritative symptoms may occur due to mucosal damage and local inflammation of the bladder endothelium, presenting with LUTS in patients suffering from COVID-19 [9].

This study aimed to analyze the severity of LUTS in patients infected with COVID-19 and to identify the correlation between the severity of these symptoms and the prognosis of the disease.

## Materials and methods

Study design and setting

After obtaining informed consent from the patients and receiving approval from the institutional ethical committee, the current prospective and observational study was carried out from June 2021 to January 2022 on patients admitted to the COVID care unit of the Department of Urology at the SMS Medical College, a tertiary care teaching hospital in Rajasthan.

Inclusion criteria

The inclusion criteria were as follows: patients more than 18 years of age, those with normal renal function tests, normal urine analysis, and microscopy and cultures, and, in male patients, normal serum prostate-specific antigen (PSA), The patient was considered to have COVID-19 if a PCR on the nasal swab returned positive for SARS-CoV-2 and there were pertinent signs on high-resolution CT (HRCT) of the chest.

Exclusion criteria

The exclusion criteria were as follows: patients with a history of previous pelvic surgical intervention or radiotherapy, and those with a history of or known cases of benign prostatic enlargement, carcinoma prostate, prostatitis, urethral stricture, bladder outlet obstruction, neurogenic bladder, acute kidney injury, or urinary tract infection; those who had ever been admitted to the ICU and given massive intravenous hydration (>3 liters) and drugs affecting LUTS (antidepressants, antihistamines, bronchodilators, non-urinary anticholinergics, sympathomimetics, and diuretics).

Procedure

Overactive bladder, also called OAB, causes a frequent and sudden urge to urinate that may be difficult to control. After obtaining informed consent, the clinical details of the patients were recorded on the day of admission. The sample population was evaluated using the OAB symptom scoring system [10] for current and pre-COVID-19 scenarios for LUTS. This system has four individual symptom scores ranging from 0 to 15 based on frequency, nocturia, urinary urgency, and urge incontinence. A questionnaire regarding patients' urinary tract symptoms was recorded in the proforma. Each symptom was scored as follows - frequency: 0-2 points, nocturia: 0-3 points, urgency: 0-5 points, and urge incontinence: 0-5 points. A cumulative score of symptoms was noted for the patient's symptoms. The patients were then classified into three groups according to their severity based on the OAB symptom scoring system, as follows: 3-5: mild, 6-11: moderate, and >12: severe (Table 1). Similarly, patients were also classified into three groups based on the CT severity scoring system for lung involvement due to COVID-19 infection, as follows: <8/24: mild, 9-15/24: moderate, and >15/24: severe [11]. The three lung lobes on the right and two lobes on the left were individually assessed, and the percentage involvement of the lobe was noted based on visual assessment. Visual severity scoring of CT chest was classified as follows: score 1: <5% area involved, score 2: 5-25% area involved, score 3: 25-50% area involved, score 4: 50-75% area involved, and score 5: >75% area involved, giving a total score of 25. A CT severity score was assigned out of 25 based on the percentage area involved in each of the five lobes. From the obtained data, the incidence and severity of LUTS in COVID-19 patients were assessed and a correlation analysis was done with pulmonary involvement [12]. The number of days required for critical care and oxygen inhalation was also noted. The CT severity score and other urological symptoms were also recorded. Patients were followed up for up to two weeks after discharge.

**Table 1:** Overactive bladder symptom score questionnaire

Questionnaire items	Frequency	Score
How many times do you typically urinate from waking up in the morning until sleeping at night? (Frequency)	≤7	0
	8-14	1
	≥15	2
How many times do you typically wake up to urinate from sleeping at night until waking up in the morning? (Nocturia)	0	0
	1	1
	2	2
	≥3	3
How many times do you have a sudden desire to urinate, which is difficult to defer? (Urgency)	Not at all	0
	Less than once a week	1
	Once a week or more	2
	About once a day	3
	2-4 times a day	4
	5 times a day or more	5
How often do you leak urine because you cannot defer the sudden desire to urinate? (Urge incontinence)	Not at all	0
	Less than once a week	1
	Once a week or more	2
	About once a day	3
	2-4 times a day	4
	5 times a day or more	5

Statistical analysis

The sample size was calculated based on a previous study by Dhar et al. [13]. The alpha error used for this analysis was 0.05. Using a table of trade-offs for any combination of sample size (n) and power, a sample size of 60 was determined to have 80% power and a 95% confidence interval. Qualitative data were expressed in the form of percentages and proportions. The chi-square test was used to analyze quantitative data expressed as mean and standard deviation (SD). A p-value <0.05 was considered statistically significant. Data obtained were analyzed using IBM SPSS Statistics version 16.0 (IBM Corp., Armonk, NY).

## Results

A total of 82 patients with LUTS during the study period were included in the study. The mean age of the patients was 62.54 years; 47 (57.32%) were male and 35 (42.68%) were female. Urinary frequency (97.56%) was the most common symptom, followed by nocturia (81.70%) and urgency (67.07%) (Table 2).

**Table 2:** Demographic details and types of LUTS (n=82) LUTS: lower urinary tract symptoms

Variables	Values
Mean age, years	62.54
Gender, n (%)	
Male	47 (57.32%)
Female	35 (42.68%)
Types of LUTS, n (%)	
Frequency	80 (97.56%)
Nocturia	67 (81.70%)
Urgency	55 (67.07%)
Urinary incontinence	36 (43.9%)

**Figure 1:**
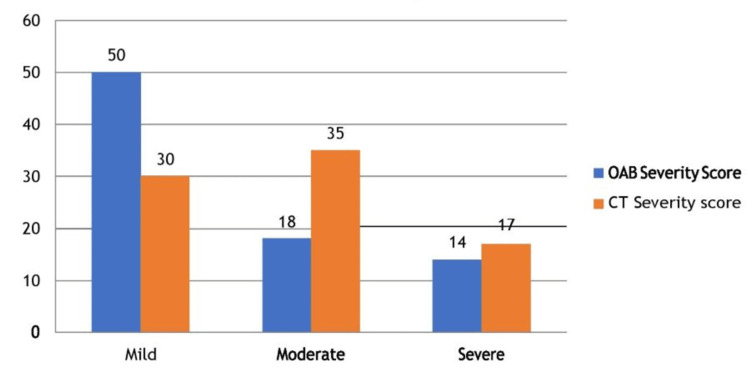
Distribution of patients as per OAB and CT severity scores CT: computed tomography; OAB: overactive bladder

As per the OAB severity score, 50 patients were in the mild category followed by 18 in the moderate and 14 in severe. CT severity score showed 30 patients in the mild category, while 35 and 17 patients were in the moderate and severe categories respectively (Figure 1).

According to the correlation analysis, the duration of hospitalization was significantly longer and the requirement for oxygen was significantly more frequent with severe LUTS symptoms (p<0.05); however, there was an insignificant correlation between OAB score and CT score (p>0.05) (Table 3).

**Table 3:** Correlation of LUTS with oxygen requirement, hospital stay, and CT score *Statistically significant CT: computed tomography; LUTS: lower urinary tract symptoms; OAB: overactive bladder

Variable	Median (range)	P-value
OAB severity score		
Male	9 (6-14)	
Female	9 (5-12)	
Correlation of severe LUTS		
Oxygen requirement (l)	13 (6-17)	<0.05*
Hospital stay (days)	12 (8-20)	<0.05*
CT score (thorax)	11 (3-23)	>0.05

## Discussion

LUTS commonly involve daytime urinary urgency, nocturia, voiding disturbances, and urinary incontinence. COVID-19 has been shown to decrease immunity in patients and now various studies have also observed an increased prevalence of LUTS in patients suffering from COVID-19. Kaya et al. have reported that LUTS might be one of the various initial symptoms of COVID-19 [14]. Mumm et al. conducted a case series to evaluate urinary symptoms as clinical features in 57 COVID-19-infected patients [5]. In acute bacterial infection of the prostate, there is an increase in voiding symptoms that are secondary to obstruction in the prostatic urethra. Also, bacterial infections of the prostate can cause an increase in PSA levels above normal. However, a viral infection of the prostate is very rare [15,16]. Studies on viral infections of the prostate in the literature are limited to case reports [16,17]. In the present study, although the base PSA values were not known, it was observed that PSA values did not exceed the normal value. In fact, even when there was a significant increase in the patient's voiding symptoms, the PSA values remained within the normal range, indicating that COVID-19 infection did not cause inflammation in the prostate.

In the present study, urinary frequency (97.56%) was the most common LUTS, followed by nocturia in COVID-19 patients. Similar results were reported by Dhar et al. in a case series conducted at a tertiary care COVID-19 unit, which found that urinary frequency (85%) of ≥13 episodes/24 hours and nocturia (87%) ≥4 episodes/night were the most common urological complaints [13]. These findings led them to identify the association of COVID-19 infection with cystitis. Another study has also shown a correlation between COVID-19 and the development of acute kidney injury, infection, and mortality (5.3%) [18]. This study observed that patients had a remarkable increase in signs of kidney dysfunction, including a 59% increase in proteinuria, a 44% increase in haematuria, a 14% increase in the level of blood urea nitrogen, and a 10% increase in serum creatinine levels following COVID-19 infection.

The present study showed an insignificant correlation between the OAB score and the CT severity score. In a prospective study involving 94 cases of COVID-19 infection, Can et al. found that LUTS scores were higher in the elderly, but disease severity did not correlate with LUTS scores [19]. In the present study, the average age of the patients was more than 60 years.

In the present study, the length of hospitalization was significantly longer and oxygen needs were also significantly more frequent with severe LUTS in COVID-19 patients. Karabulut et al. conducted a prospective study including 63 male COVID-19 patients who were over 40 years of age. According to the International Prostate Symptom Score (IPSS) grading of LUTS, patients with high IPSS had longer hospitalization, an increased need for critical care, and an increased mortality rate. Hence, they concluded that benign prostate hypertrophy-related LUTS can be used as a prognostic tool for COVID-19 infection [20].

## Conclusions

The findings of our study suggest that patients with COVID-19 may develop or experience de novo LUTS, notably storage symptoms. The length of hospital stay for COVID-19 patients may also be influenced by the severity of their LUTS. Therefore, medical professionals should consider COVID-19-related bladder dysfunctions such as de novo LUTS as part of COVID-19 symptomatology.
